# Protective Effects of a Hyaluronan-Binding Peptide (P15-1) on Mesenchymal Stem Cells in an Inflammatory Environment

**DOI:** 10.3390/ijms22137058

**Published:** 2021-06-30

**Authors:** Thorsten Kirsch, Fenglin Zhang, Olivia Braender-Carr, Mary K. Cowman

**Affiliations:** 1 Department of Orthopaedic Surgery, New York University Grossman School of Medicine, New York, NY 10010, USA; fenglin.zhang@nyulangone.org (F.Z.); mary.cowman@nyu.edu (M.K.C.); 2 Department of Biomedical Engineering, New York University Tandon School of Engineering, New York, NY 10010, USA; obc208@nyu.edu

**Keywords:** mesenchymal stem cells, hyaluronan, peptide, inflammation, articular cartilage

## Abstract

Mesenchymal stem cells (MSCs) obtained from various sources, including bone marrow, have been proposed as a therapeutic strategy for the improvement of tissue repair/regeneration, including the repair of cartilage defects or lesions. Often the highly inflammatory environment after injury or during diseases, however, greatly diminishes the therapeutic and reparative effectiveness of MSCs. Therefore, the identification of novel factors that can protect MSCs against an inflammatory environment may enhance the effectiveness of these cells in repairing tissues, such as articular cartilage. In this study, we investigated whether a peptide (P15-1) that binds to hyaluronan (HA), a major component of the extracellular matrix of cartilage, protects bone-marrow-derived MSCs (BMSCs) in an inflammatory environment. The results showed that P15-1 reduced the mRNA levels of catabolic and inflammatory markers in interleukin-1beta (IL-1β)-treated human BMSCs. In addition, P15-1 enhanced the attachment of BMSCs to HA-coated tissue culture dishes and stimulated the chondrogenic differentiation of the multipotential murine C3H/10T1/2 MSC line in a micromass culture. In conclusion, our findings suggest that P15-1 may increase the capacity of BMSCs to repair cartilage via the protection of these cells in an inflammatory environment and the stimulation of their attachment to an HA-containing matrix and chondrogenic differentiation.

## 1. Introduction

Mesenchymal stem cells (MSCs), including bone-marrow-derived mesenchymal stem cells (BMSCs), are essential for the repair of many tissues, including the repair of cartilage defects or lesions. In addition, MSCs have been shown to exhibit anti-inflammatory properties. Therefore, the use of MSCs in various diseases and tissue repair/regeneration is a matter of intensive research [1,2,3,4]. For example, the MSC transplantation in chronic wounds has been suggested. However, studies showed low success of improved wound healing after injection of adipose-derived MSCs and/or BMSCs, most likely due to the harsh injury microenvironment that is infiltrated by inflammatory and catabolic cytokines and reactive oxygen species [5,6,7]. Furthermore, previous studies have shown that prolonged exposure of MSCs to interleukin (IL)-1β, the main cytokine present in the injured or osteoarthritic joints, results in an inhibition of chondrogenic differentiation and increased expression of matrix-degrading enzymes by MSCs [8,9,10]. 

The extracellular matrix (ECM) has been shown to play a crucial role for MSC maintenance, homeostasis, and differentiation. Within the ECM, the glycosaminoglycan hyaluronan (HA) acts as a centrally important molecule. HA is tethered to the cell surface by binding to the integral membrane protein CD44 [11,12,13]. The molecular weight distribution of HA is polydisperse. In healthy tissues, the molecular weight is high, generally with averages of approximately 2–8 MDa [14]. In this high molecular weight form, a single HA molecule can simultaneously bind and cluster multiple CD44 receptors [15]. Under inflammatory conditions, HA can be degraded by reactive oxygen and nitrogen species or by enzymatic cleavage, which can result in the de-clustering of CD44 and altered intracellular signaling [15,16]. Thus, the HA molecular weight is important to CD44 clustering and signaling, and multiple studies have documented that fragmented HA can act as a danger sensor, leading to inflammatory or defensive responses [17,18,19,20].

HA has been shown to be an integral part of the stem cell niche and plays important roles in MSC function and differentiation [12,21,22,23,24]. Since HA and its signaling play key roles in cellular function and differentiation, the amount of HA produced by cells has to be highly regulated. Several studies have shown that MSCs cultured in a monolayer produce little HA. Certain growth factors, however, such as fibroblast growth factor (FGF), stimulate the production of HA by MSCs [25,26,27]. On the other hand, cells which are embedded in an ECM, such as chondrocytes, produce large amounts of HA and have a pericellular coat rich of HA [28,29,30]. For example, MSCs, before undergoing chondrogenesis, express CD44 but produce low amounts of HA. After condensation and initiation of chondrogenic differentiation, the cells markedly increase the production of HA [31]. 

In addition, the interactions of HA with its various receptors control the effect of HA on cells. For example, the HA-binding protein, named receptor for hyaluronan-mediated motility (RHAMM), has been shown to stimulate the migration of tumor cells [32], fibroblasts [33], and macrophages [34] and to contribute to myofibroblast differentiation [35] in remodeling tissue. Furthermore, the interaction of HA with RHAMM and CD44 was shown to enhance stem cell migration and proliferation [36], but also inflammatory and fibrotic processes [34,37]. In addition, a previous study showing that HA/RHAMM interactions result in the inhibition of adipogenic differentiation of MSCs suggests that HA/RHAMM interactions may also affect the differentiation of MSCs [38]. RHAMM is therefore a therapeutic target, and peptides that modulate interactions between HA and RHAMM can reduce inflammation and fibrotic processes [34,35,37,39]. Candidate therapeutic peptides that target HA–RHAMM interactions can be identified not only by rational design, based on HA binding sequences in RHAMM, but also by using unbiased peptide library screening methods such as phage display [39,40].

The amino acid sequences of mouse RHAMM that bind HA are 401–411 (KQKIKHVVKLK) and 423–432 (KLRSQLVKRK) [41]. The HA-binding sequences contain a BX_7_B motif, where B is R or K amino acids in RHAMM, X is not acidic amino acid, and X should include at least one basic amino acid [42]. Our study utilizes a 15-mer peptide, STMMSRSHKTRSHHV, called P15-1, that was discovered using a phage display peptide library to identify HA-binding peptides with homology to the HA-binding site of RHAMM [37]. P15-1 binds HA, blocks cell mobility and has a BX_7_B motif similar to RHAMM. It competes with RHAMM for HA binding. P15-1 promotes skin wound healing without fibrosis [37,40]. P15-1 is anti-inflammatory in interleukin-1beta (IL-1β)-stimulated human articular chondrocytes [43] and is reported to promote the non-fibrotic repair of a full thickness injury to articular cartilage in a rabbit model [44].

Since repair processes involving MSCs after tissue injury or trauma occur in an inflammatory environment that has been shown to be deleterious for stem-cell-mediated repair, the goal of this study was to determine whether P15-1 as a potential anti-inflammatory agent can modulate deleterious effects of an inflammatory environment on MSCs. 

## 2. Results

### 2.1. P15-1 Decreases Catabolic and Inflammatory Events in Human BMSCs

**Figure 1:**
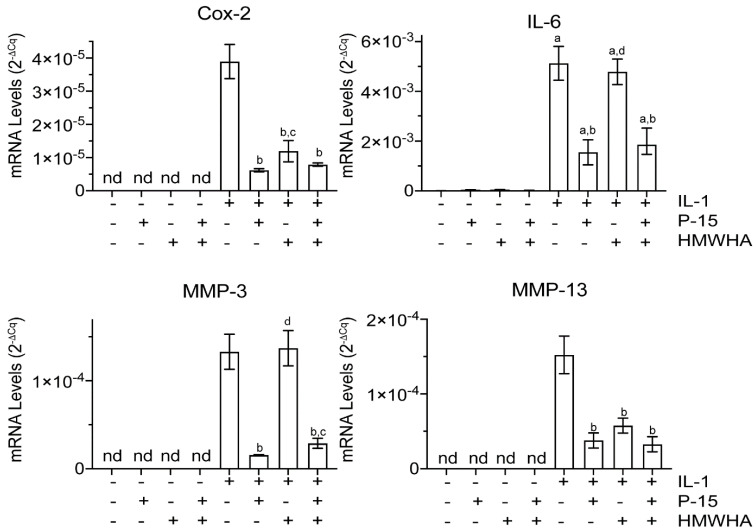
mRNA levels of catabolic markers in vehicle–treated or interleukin–1beta (IL–1β)-treated human bone–marrow–derived mesenchymal stem cells (BMSCs) cultured in the absence or presence of P15–1 and high–molecular–weight hyaluronan (HMWHA). Human BMSCs were serum–starved for 24 h followed by treatment with vehicle or IL–1β in the absence or presence of P15–1, HMWHA, or P15–1 and HMWHA for 48 h. The mRNA levels of catabolic markers (Cox–2 and interleukin (IL)–6) and metalloproteinases (MMP–3 and MMP–13) were determined by real–time PCR using SYBR Green and normalized to the level of 18S RNA. Data were obtained from triplicate PCRs using RNA from 3 different cultures. Values are the mean ± standard deviation (SD). ^a^ *p* < 0.01 vs. vehicle–treated cells; ^b^ *p* < 0.01 vs. IL–1β–treated cells; ^c^ *p* < 0.05 vs. IL-1β/P15–1–treated cells; ^d^ *p* < 0.01 vs. IL–1β/P15–1–treated cells; nd, not detected.

The treatments of human BMSCs with IL-1β at various concentrations (2, 5, and 10 ng/mL) for 24 h resulted in the expression of catabolic and inflammatory markers, including cyclooxygenase-2 (Cox-2) and interleukin-6 (IL-6), as well as matrix metalloproteinases (MMPs), MPP-3, and MMP-13 with IL-1β at a concentration of 10 ng/mL inducing the highest mRNA levels of these markers (data not shown). Based on these findings, we treated human BMSCs with 10 ng/mL IL-1β in all experiments. In the absence of IL-1β, human BMSCs expressed Cox-2, IL-6, MMP-3, and MMP-13 at very low or undetectable levels, whereas in the presence of IL-1β the mRNA levels of these markers were highly increased. P15-1 alone markedly reduced the mRNA levels of the catabolic markers Cox-2, IL-6, MMP-3, and MMP-13 in IL-1β-treated human BMSCs. The treatment of BMSCs with a high-molecular-weight HA (HMWHA) reduced the mRNA levels of Cox-2 and MMP-13 in IL-1β-treated BMSCs, but to a lesser degree than P15-1. IL-6 and MMP-3 mRNA levels in IL-1β-treated BMSCs were not affected by HMWHA treatment. When P15-1 was pre-mixed with HMWHA and then the mixture was used to treat human BMSCs in the presence of IL-1β, P15-1 together with HMWHA reduced the mRNA levels of Cox-2, IL-6, MMP-3, and MMP-13 to levels similar to P15-1 alone (Figure 1).

### 2.2. P15-1 Decreases the mRNA Levels of Inflammatory Receptors TLR2 and TLR4 in IL-1β-Treated Human BMSCs

**Figure 2:**
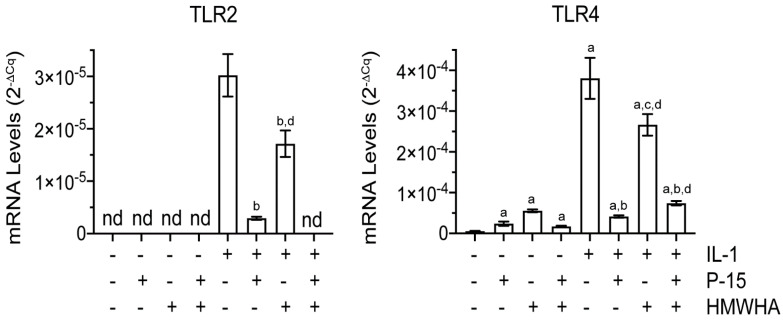
mRNA levels of Toll—like receptor 2 (TLR2) and Toll—like receptor 4 (TLR4) in vehicle—treated or IL—1β—treated human BMSCs cultured in the absence or presence of P15—1 and HMWHA. Human BMSCs were serum—starved for 24 h followed by treatment with vehicle or IL—1β in the absence or presence of P15—1, HMWHA, or P15—1 and HMWHA for 48 h. The mRNA levels of TLR2 and TLR4 were determined by real—time PCR using SYBR Green and normalized to the level of 18S RNA. Data were obtained from triplicate PCRs using RNA from 3 different cultures. Values are the mean ± SD. ^a^ *p* < 0.01 vs. vehicle–treated cells; ^b^ *p* < 0.01 vs. IL–1β-treated cells; ^c^ *p* < 0.05 vs. IL–1β-treated cells; ^d^ *p* < 0.01 vs. IL-1β/P15–1–treated cells; nd, not detected.

BMSCs expressed the inflammatory receptor Toll-like receptor 4 (TLR4) at low levels, whereas the expression of Toll-like receptor 2 (TLR2) was not detectable. The mRNA levels of TLR2 and TLR4 were markedly increased in IL-1β-treated BMSCs. P15-1 or HMWHA treatment, when used separately, reduced the mRNA levels of TLR2 and TLR4 in IL-1βtreated BMSCs with P15-1 being more effective than HMWHA. P15-1 together with HMWHA reduced the mRNA levels of TLR2 to non-detectable levels in IL-1β-treated cells, whereas P15-1 together with HMWHA was less effective in reducing the mRNA levels of TLR4 than P15-1 alone (Figure 2). 

### 2.3. P15-1 Affects the mRNA Levels of HA Receptor CD44 and RHAMM Receptor and TSG-6 in Human BMSCs 

**Figure 3:**
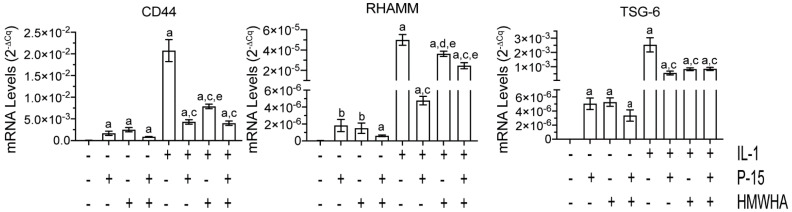
mRNA levels of HA receptors HA–binding proteins, CD44, RHAMM, and TSG–6 in vehicle–treated or IL–1β–treated human BMSCs cultured in the absence or presence of P15–1 and HMWHA. Human BMSCs were serum–starved for 24 h followed by treatment with vehicle or IL–1β in the absence or presence of P15–1, HMWHA, or P15–1 and HMWHA for 48 h. The mRNA levels of CD44, receptor for hyaluronan–mediated motility (RHAMM), and tumor necrosis factor–stimulated gene–6 (TSG–6) were determined by real–time PCR using SYBR Green and normalized to the level of 18S RNA. Data were obtained from triplicate PCRs using RNA from 3 different cultures. Values are the mean ± SD. ^a^ *p* < 0.01 vs. vehicle–treated cells; ^b^ *p* < 0.05 vs. vehicle–treated cells; ^c^ *p* < 0.01 vs. IL-1β–treated cells; ^d^ *p* < 0.05 vs. IL–1β–treated cells; ^e^ *p* < 0.01 vs. IL–1β/P15–1–treated cells.

P15-1, HMWHA, or P15-1 together with HMWHA increased the mRNA levels of the HA receptors CD44 and RHAMM compared to vehicle treatment. The IL-1β treatment increased the mRNA levels of CD44 and RHAMM to levels markedly higher than the levels of P15-1-, HMWHA-, or P15-1/HMWHA-treated cells. P15-1 or HMWHA reduced the mRNA levels of CD44 and RHAMM in IL-1β-treated BMSCs with P15-1 being more effective than HMWHA. P15-1 together with HMWHA was less effective in reducing RHAMM mRNA levels than P15-1 alone. Tumor necrosis factor-stimulated gene-6 (TSG-6) is a protein that binds to HA and is able to cross-link HMWHA [45]. In addition, this protein is upregulated in many cell types in inflammatory disease states [46,47,48]. Here, we showed that the mRNA levels of TSG-6 in vehicle-treated BMSCs were very low. P-15-1 alone, HMWHA alone, and P15-1 together with HMWHA increased the mRNA levels of TSG-6 in vehicle-treated BMSCs to a similar degree. TSG-6 mRNA levels were also increased by IL-1β treatment. IL-1β increased the mRNA levels of TSG-6 to levels markedly higher than those in P15-1-, HMWHA-, or P15-1/HMWHA-treated cells. P15-1 alone, as well as HMWHA or P15-1 together with HMWHA, reduced the mRNA levels of TSG-6 in IL-1β-treated BMSCs to a similar degree (Figure 3). 

### 2.4. P15-1 Alters the mRNA Levels of HA-Producing and -Degrading Enzymes in BMSCs

**Figure 4:**
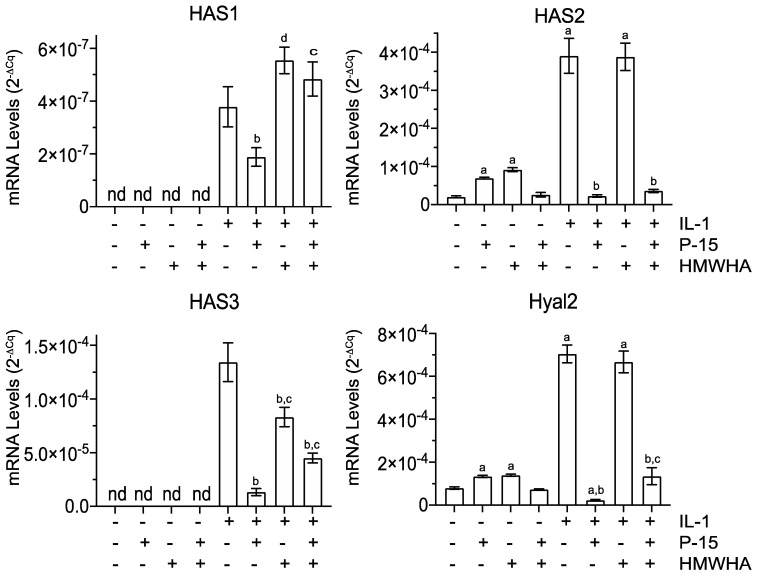
mRNA levels of HA synthase enzymes (HAS1, HAS2, and HAS3) and the HA–degrading enzyme Hyal2 in vehicle–treated or IL–1β–treated human BMSCs cultured in the absence or presence of P15–1 and HMWHA. Human BMSCs were serum–starved for 24 h followed by treatment with vehicle or IL–1β in the absence or presence of P15–1, HMWHA, or P15–1 and HMWHA for 48 h. The mRNA levels of HAS1, HAS2, HAS3, and Hyal2 were determined by real–time PCR using SYBR Green and normalized to the level of 18S RNA. Data were obtained from triplicate PCRs using RNA from 3 different cultures. Values are the mean ± SD. ^a^ *p* < 0.01 vs. vehicle–treated cells; ^b^ *p* < 0.01 vs. IL–1β treated cells; ^c^ *p* < 0.01 vs. IL–1β/P15–1-treated cells; ^d^ *p* < 0.05 vs. IL–1β-treated cells; nd, not detected.

IL-1β treatment also altered mRNA levels for proteins important in HA synthesis and degradation. We observed the mRNA levels for the hyaluronan synthases (HAS), HAS1, HAS2, and HAS3, to be increased by IL-1β. The mRNA levels of HAS1 in IL-1β-treated BMSCs were markedly lower than the mRNA levels of HAS2 and HAS3. In addition, the mRNA level for the degradative hyaluronidase 2 enzyme (Hyal2) was increased by IL-1β treatment. Whereas HAS2 was increased by P15-1 alone or HMWHA alone in vehicle-treated BMSCs but not by P15-1 together with HMWHA, HAS1 and HAS3 expressions were not detectable in vehicle-treated, P15-1-treated, HMWHA-treated, or P15-1/HMWHA-treated cells. Hyal2 mRNA levels were increased by P15-1 or HMWHA in vehicle-treated BMSCs, but not by P15-1 together with HMWHA. In IL-1β-treated BMSCs, P15-1 decreased the mRNA levels of HAS1, HAS2, HAS3, and Hyal2. P15-1 together with HMWHA decreased the mRNA levels of HAS2, HAS3, and Hyal2 to levels similar to P15-1 alone or to a lesser degree than P15-1 alone. The HAS1 mRNA levels were increased in IL-1β-treated BMSCs in the presence of HMWHA or P15-1 together with HMWHA compared to in IL-1β-treated cells (Figure 4).

### 2.5. P15-1 Enhances Viability of IL-1β-Treated BMSCs

**Figure 5:**
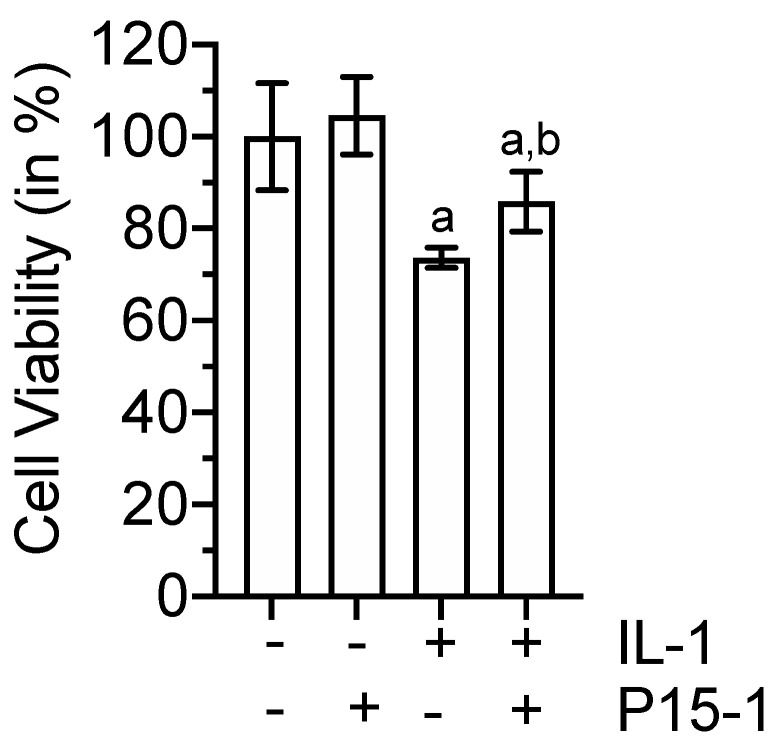
Cell viability of BMSCs cultured in the absence or presence of IL–1β and P15–1 for 48 h. Cell viability was determined using the CCK–8 viability assay. The absorbance was measured at 460 nm, and the value obtained for vehicle–treated BMSCs was set to 100%. Data were obtained from three different experiments. Values are the mean ± SD. ^a^ *p* < 0.05 vs. vehicle–treated BMSCs; ^b^ *p* < 0.05 vs. IL–1β–treated BMSCs.

The Treatment of BMSCs with 10 ng/mL IL-1β for 48 h reduced the viability of these cells by ~25% compared to the vehicle treatment or P15-1 treatment of BMSCs. The P15-1 treatment increased the cell viability of IL-1β-treated BMSCs by ~10% to a level of 85% compared to the vehicle treatment of cells (Figure 5).

### 2.6. P15-1 Enhances the Attachment of BMSCs to HA-Coated Tissue Culture Dishes

**Figure 6:**
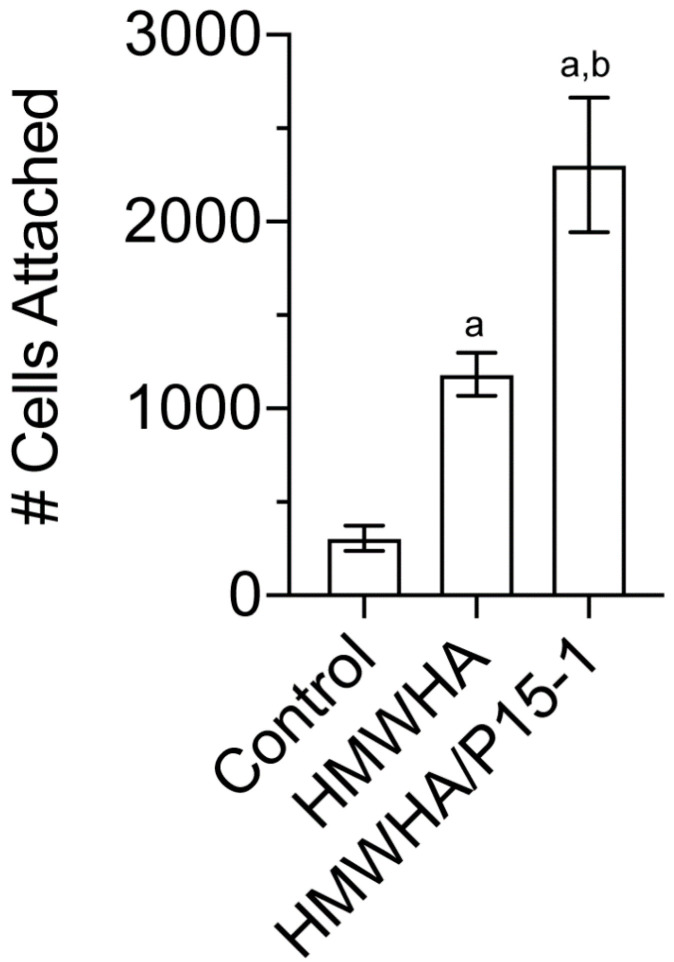
Numbers of human BMSCs attached to tissue culture dish (control), HMWHA–coated tissue culture dish (HMWHA), and tissue culture dish coated with HMWHA together with P15–1 (HMWHA/P15–1) after 20 min incubation of a suspension containing 1 × 10^5^ cells at 37 °C. The number of crystal violet stained cells attached to the dishes was counted. Data are presented as mean ± SD from three different experiments. ^a^ *p* < 0.01 vs. control; ^b^ *p* < 0.01 vs. HMWHA.

For tissue repair to occur, BMSCs need to first attach to the site of injury and then undergo differentiation. Since HA is an important extracellular component of the stem cell niche and is present at the repair site [12,21,22,23,24], we determined the attachment of BMSCs to HMWHA-coated tissue culture plates in the absence or presence of P15-1. Figure 6 displays the average numbers of human BMSCs attached to a tissue culture dish, HMWHA-coated tissue culture dish, and tissue culture dish coated with HMWHA together with P15-1. More BMSCs attached to HMWHA-coated tissue culture dishes than to uncoated dishes. The number of attached cells was further increased on tissue culture dishes that were coated with HMWHA together with P15-1 (Figure 6).

### 2.7. P15-1 Enhances the Chondrogenic Differentiation of C3H10T1/2 Cells

**Figure 7:**
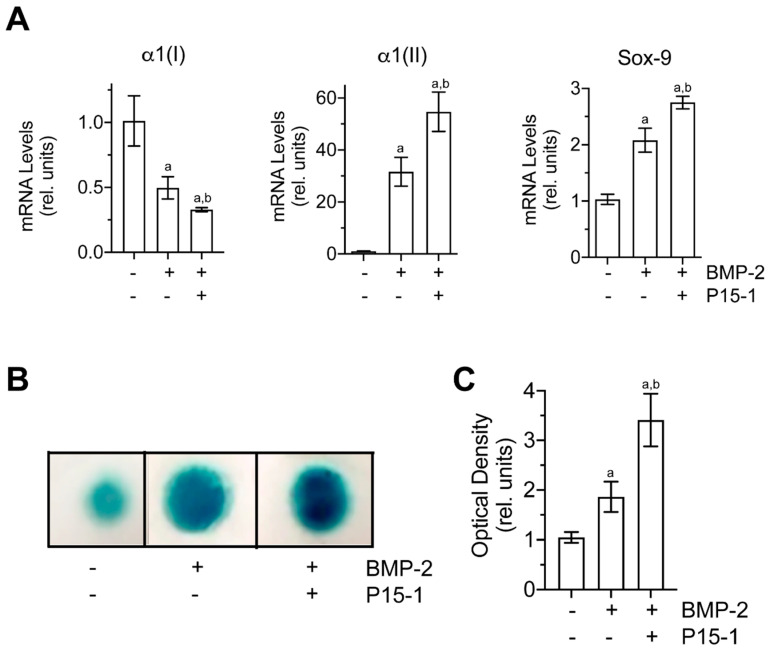
(**A**) mRNA levels of type I collagen (α1(I)), type II collagen (α1(II)), and Sox–9 in C3H10T1/2 micromasses cultured in the absence or presence of BMP–2 and P15–1. C3H10T1/2 cells were cultured in micronasses in the absence or presence of BMP–2 and P15–1 for 3 days. The mRNA levels of type I collagen (α1(I)), type II collagen (α1(II)), and Sox–9 were determined by real-time PCR using SYBR Green and normalized to the level of 18S RNA. Data were obtained from triplicate PCRs using RNA from 3 different cultures. Data are expressed as relative units with the mRNA levels of the vehicle–treated cells set as 1. Values are the mean ± SD. (**B**) Alcian blue staining of C3H10T1/2 micromasses cultured in the absence or presence of BMP–2 and P15–1 for 3 days. (**C**) Optical density of the stained Alcian blue micromases analyzed by ImageJ. Data are expressed as relative units with the values of vehicle–treated micromasses set as 1. ^a^ *p* < 0.01 vs. vehicle-treated cells; ^b^ *p* < 0.01 vs. BMP-2–treated cells.

The multipotential murine C3H/10T1/2 MSC line has been shown to be a good alternative source of BMSCs for investigating chondrogenic differentiation when cultured in micromasses in the presence of bone morphogenetic protein-2 (BMP-2) [49]. C3H10T1/2 micromasses cultured in the presence of BMP-2 showed increased mRNA levels of the chondrocyte-specific genes, Sox-9 and type II collagen, and reduced mRNA levels of type I collagen compared to vehicle-treated micromasses (Figure 7A). In addition, BMP-2-treated micromasses showed increased alcian blue staining compared to vehicle-treated micromasses, indicating increased chondrogenesis in BMP-2-treated micromasses (Figure 7B). P15-1 further increased the mRNA levels of Sox-9 and type II collagen as well as alcian blue staining in BMP-2-treated micromasses (Figure 7A,B). In addition, P15-1 further decreased the mRNA levels of type I collagen in BMP-2-treated micromasses (Figure 7A). P15-1 in the absence of BMP-2 had no effect on the micromasses (data not shown). These findings indicate that P15-1 enhances chondrogenic differentiation in BMP-2-treated C3H10T1/2 micromasses. 

## 3. Discussion

Tissue repair by MSCs often occurs in an inflammatory environment, and this harsh inflammatory injury environment has been suggested to inhibit the repair process by MSCs. For example, previous studies have shown that an inflammatory environment inhibits chondrogenic differentiation of BMSCs [9,10]. In this study, we showed that IL-1β, one of the main inflammatory cytokines in the osteoarthritic or injured joint [50], stimulates the expression of catabolic markers, Cox-2 and IL-6, and MMP-3 and MMP-13, in human BMSCs. In addition, we showed that the 15mer HA-binding peptide, P15-1, inhibited the expression of Cox-2, IL-6, MMP-3, and MMP-13 in IL-1β-treated BMSCs. In a previous study, we showed that P15-1 also inhibits catabolic and inflammatory events in IL-1β-treated articular chondrocytes [51]. The findings presented in this study and in our previous study suggest that P15-1 may act as an anti-inflammatory factor that may reduce inflammation in the osteoarthritic joint or injured joint. Based on these findings, it can be speculated that P15-1 by reducing the inflammatory injury environment may enhance the repair process of the injured tissue by MSCs.

P15-1 also enhanced the attachment of BMSCs to HMWHA-coated tissue culture dishes and the chondrogenic differentiation of the murine C3H/10T1/2 MSC line when cultured in a micromass culture in the presence of BMP-2. Previous studies have shown that the extracellular components of the niche play a crucial role in MSC attachment and differentiation [29]. For example, it has been demonstrated that HA in the extracellular niche stimulates chondrogenic differentiation of MSCs [21,22]. Clustering of CD44 has been shown to play a critical role in the stimulation of chondrogenesis by HMWHA. The clustering of CD44 by HMWHA has been shown to stimulate ERK signaling in MSCs and ultimately the expression and activity of Sox-9, a transcription factor essential for chondrogenesis [23]. In addition, HA-mediated CD44 clustering can prevent or enhance the interactions of various factors with their receptors, thereby controlling MSC attachment and chondrogenic differentiation [29]. It can be speculated that P15-1 stabilizes the interaction of HA with CD44 and its ability to cluster CD44, resulting in the increased attachment of MSCs to HA and the stimulation of chondrogenic differentiation of MSCs. 

Our findings show that vehicle-treated human BMSCs express TSG-6 at low levels. P15-1, HMWHA, and P15-1 together with HMHA increased the mRNA levels of TSG-6 in vehicle-treated cells. Furthermore, when cultured in the presence of IL-1β human BMSCs further increased the expression levels of TSG-6. Our findings are consistent with previous findings showing a very low expression of TSG-6 by MSCs in monolayer and a marked increase of TSG-6 expression by MSCs when cultured in an inflammatory environment [52,53]. TSG-6 has been shown to have broad ranging effects, including suppression of inflammation, the reduction of tissue injury and disease indices, and the enhancement of healing and repair processes [53,54]. The mechanisms of actions of TSG-6 are still poorly understood. However, some of the TSG-6 effects are most likely mediated via its regulation of HA/CD44 interactions, its ability to cross-link HA and stabilize the pericellular matrix, and its ability to bind to various chemokines [52,54]. Our study, however, demonstrates that the increased expression of TSG-6 in IL-1β-treated BMSCs was not able to prevent the increase of the expression of catabolic markers. It is likely that the prolonged exposure of BMSCs to IL-1β eventually leads to the upregulation of various catabolic genes, resulting in a loss of the anti-inflammatory effects and repair capacity of BMSCs despite the high expression of TSG-6. In addition, based on the increased mRNA levels of the HA-degrading enzyme Hyal2, it can be speculated that the IL-1β treatment may have led to increased HA degradation and, as a consequence, a less protective pericellular matrix around BMSCs.

The findings shown in this study were obtained with 10 ng/mL IL-1β. This concentration is higher than the concentration of IL-1β found in the synovial fluid from a joint after injury. However, we have obtained similar results with lower concentrations of IL-1β (data not shown). Ten ng/mL of IL-1β, however, showed the most profound effects on BMSCs in monolayer cultures. Furthermore, we chose a long exposure of IL-1β (48 h) to BMSCs to mimic the prolonged harsh inflammatory environment for MSCs in an injured or osteoarthritic joint or other injured tissue. In contrast, short exposure of MSCs to inflammatory factors, such as IL-1, which has been shown to enhance the anti-inflammatory potential of MSCs [55], may be sufficient for the upregulation of anti-inflammatory factors, such as TSG-6, but not for the pronounced upregulation of catabolic genes, ultimately resulting in an increased anti-inflammatory potential of MSCs. 

Previously, we have shown that P15-1 together with HMWHA is more effective in inhibiting catabolic events in articular chondrocytes than P15-1 or HMWHA alone [43]. In this study, we showed that P15-1 alone was similar or even more effective than the combined treatment of IL-1β-treated BMSCs with P15-1 and HMWHA. A previous study has shown that umbilical cord stem cells exposed to inflammatory cells produce an HA-rich pericellular matrix, which was stabilized by TSG-6 and versican and allow the stem cells to survive in the inflammatory environment [13]. Therefore, it is possible that additional HMWHA treatment of BMSCs does not further improve a HA-containing pericellular matrix formed by these cells in an inflammatory environment. P15-1 may further help to stabilize this pericellular matrix, and/or the peptide may work by another mechanism independent of its effect on HA. Future experiments need to explore the mechanisms of how P15-1 protects MSCs in an inflammatory environment. 

In conclusion, our study demonstrates that an HA-binding peptide, P15-1, decreased the expression of catabolic markers, such as Cox-2 and IL-6, and the expression of matrix-degrading matrix metalloproteinases, MMP-3 and MMP-13 in human BMSCs cultured in an inflammatory environment (in the presence of IL-1β). In addition, the peptide decreased the expression of the HA-degrading enzyme, Hyal2, and decreased the expression of the inflammatory receptors TRL2 and TLR4 in IL-1β-treated human BMSCs. Especially, TLR4 signaling has been shown to result in the expression of pro-inflammatory mediators by MSCs [8]. Furthermore, P15-1 enhanced the attachment of human BMSCs to an HMWHA-coated tissue culture dish and the chondrogenic differentiation of C3H10T1/2 cells. Therefore, P15-1 may provide a novel compound that may help to maintain the anti-inflammatory properties of MSCs in an inflammatory environment during tissue repair or diseases and promote the attachment, settlement, and differentiation of MSCs to an HA-containing matrix at a repair site. 

## 4. Materials and Methods

### 4.1. Reagents

P15-1 (STMMSRSHKTRSHHV) peptide having low endotoxin levels was obtained from Genscript. IL-1β was obtained from R&D Systems (Minneapolis, MN, USA). Polydisperse HA samples with an average molecular weight range of 1.01–1.8 MDa and having low endotoxin levels (≤0.004 endotoxin units (EU)/mg) were obtained from Lifecore Biomedical LLC (Chaska, MN, USA). Proteinase K was purchased from Roche Applied Bio Science (Penzberg, Germany). Media for the culture of human BMSCs were obtained from RoosterBio, Inc. (Frederick, MD, USA). All other chemicals were obtained from Sigma-Aldrich (St. Louis, MO, USA).

### 4.2. Human BMSC Culture

Pre-characterized human BMSCs from one donor were obtained from RoosterBio, Inc. (Frederick, MD, USA). All cells for experiments were maintained for up to two passages. Briefly, human BMSCs were expanded and cultured in RoosterNourish^TM^-MSC medium according to the manufacturer’s instructions. After the cells reached 70% confluency, the medium was switched to a serum-free RoosterBasal^TM^-MSC medium. After exposure of the cells to the serum-free medium for 24 h, the cells were treated with various concentrations (2, 5, and 10 ng/mL) of IL-1β in phosphate-buffered saline (PBS) and 0.1% bovine serum albumin (BSA) in the serum-free medium for 48 h. In addition, the cells were treated with 17 µg/mL P15-1 in the absence or presence of 1 mg/mL HMWHA (LifeCore, Chaska, MN, USA). Control cells were cultured in the presence of vehicle (PBS/0.1% BSA). Data were obtained from three different experiments and expressed as the mean ± standard deviation (SD).

### 4.3. Chondrogenic Differentiation of Murine Multipotential Stem Cell Line C3H10T1/2

The murine multipotential stem cell line, C3H10T1/2 clone 8, was obtained from American Type Culture Collection (ATCC; Manassas, VA, USA). Monolayer cultures were maintained in DMEM containing 10% fetal calf serum (FCS) (growth medium). To induce chondrogenesis, we used a miromass culture system, ITS  +  premix (insulin, transferrin, selenous acid, BSA, and linoleic acid; Corning, Corning, NY, USA) and bone morphogenetic protein (BMP)-2 (Peprotech, Rocky Hill, NJ, USA) using 10 µL drops of cells at 1 × 10^7^ cells/mL as described previously [56]. Micromass cultures were maintained in the growth medium with or without 100 ng/mL recombinant murine BMP-2 and P15-1 (17 µg/mL) for up to 6 days. The medium was changed every other day. Total RNA was isolated after 3 days of micromass cultures and analyzed for the mRNA levels of chondrocyte markers, Sox-9 and type II collagen, and the stem cell marker, type I collagen. In addition, alcian blue staining (1% Alcian blue (Sigma-Aldrich, St. Louis, MO, USA) in 3% glacial acetic acid solution) of the micromasses was performed to determine the degree of chondrogenic differentiation.

### 4.4. Cell Attachment Assay

To coat tissue culture wells, 48 well tissue culture plates were incubated with 1 mg/mL HMWHA (molecular weight range: 1.01–1.8 MDa; LifeCore, Chaska, MN, USA) in PBS or 1 mg/mL HMWHA together with 17 µg/mL P15-1 for 24 h at 4 °C. After washing and blocking the plates with 10 mg/mL heat-denatured BSA in PBS, human BMSCs at a concentration of 5 × 10^5^ cells/mL in a serum-free medium were added to the tissue culture wells and incubated at 37 °C for 20 min. After washing, the attached cells were fixed with ethanol and stained with 0.1% (*w*/*v*) crystal violet and counted. 

### 4.5. Reverse Transcription–PCR and Real-Time PCR Analysis

Total RNA was isolated from human BMSC cultures using a RNeasy Mini kit (Qiagen, Valencia, CA, USA). The levels of messenger RNA (mRNA) for CD44, Cox-2, HAS2, HAS3, Hyal2, IL-6, MMP-3, MMP-13, RHAMM, Sox-9, TLR-2, TLR-4,TSG-6, type I collagen (α1(I)), and type II collagen (α(II)) were quantified by real-time PCR, as previously described [41]. Total RNA was reversed transcribed into cDNA using the High Capacity cDNA synthesis kit (Applied Biosystems, Foster City, CA, USA). The cDNA was used as the template to quantify the relative content of mRNA by real-time PCR (ABI Stepone Plus; Applied Biosystems, Foster City, CA, USA), with the appropriate primers (see Table 1) and RT^2^ SYBR Green ROX FAST Mastermix (Qiagen, Valencia, CA, USA). The following PCR conditions were used as follows: 95 °C for 10 min, followed by 40 cycles of 95 °C for 10 s and 60 °C for 30 s, and 1 cycle of 95 °C for 15 s and 60 °C for 1 min. The 18S RNA was amplified at the same time and used as an internal control. The cycle threshold values for 18S RNA and the samples were measured and calculated. Transcript levels were calculated according to the equation x = 2^−ΔCq^, where ΔCq = Cq_exp_ − Cq_18S_. Analysis of the melting curves of the PCR products were performed in order to exclude the production of unspecific products or primer dimer synthesis.

### 4.6. Statistical Analysis

We analyzed the data using PRISM statistical analysis software. We performed descriptive statistics (mean, SD, medians, and interquartile ranges) for each outcome variable. We used analysis of the variance (ANOVA) to determine differences in means among three or more groups, while t tests were used to compare two groups. If there were significant differences in ANOVA, pairwise tests were conducted to assess specific differences using Tukey’s multiple comparison procedure. A *p*-value of <0.05 was used as a threshold for statistical significance.

## 5. Patents

Mary Cowman and Thorsten Kirsch are listed as inventors on a U.S. patent (patent No: US 10,449,229 B2).

## Figures and Tables

**Table 1:** The sequences of the primers used for real-time PCR.

Primer Name	Sequences (5′ to 3′)
α1(I)	F: 5′-GAGGGCCAAGACGAAGACATC-3′
	R: 5′-CAGATCACGTCATCGCACAAC-3′
α1(II)	F: 5′-GGCAATAGCAGGTTCACGTACA-3′
	R: 5′-CGATAACAGTCTTGCCCCACTT-3′
CD44	F: 5′-AGCATCGGATTTGAGACCTG-3′
	R: 5′-GTTGTTTGCTGCACAGATGG-3′
Cox-2	F: 5′-CGGTGAAACTCTGGCTAGACAG-3′
	F: 5′-RGCAAACCGTAGATGCTCAGGGA-3′
HAS1	F: 5′-ATCCTGCATCAGCGGTCCTC-3′
	R: 5′-CTGGTTGTACCAGGCCTCAAGAA-3′
HAS2	F: 5′-GAGGACGACTTTATGACCAAGAG-3′
	R: 5′-AAAGAGTGTGGTTCCAATTATTCTC-3′
HAS3	F: 5′-AGCACCTTCTCGTGCATCATGC-3′
	R: 5′-TCCTCCAGGACTCGAAGCATCT-3′
Hyal2	F: 5′-CCCAGTCTACGTCTTCACACG-3′
	R: 5′-TCCATCTCACTAAGCCCCG-3′
IL-6	F: 5′-AGACAGCCACTCACCTCTTCAG-3′
	R: 5′-TTCTGCCAGTGCCTCTTTGCTG-3′
MMP-3	F: 5′-CACTCACAGACCTGACTCGGTT-3′
	R: 5′-AAGCAGGATCACAGTTGGCTGG-3′
MMP-13	F: 5′-CCAGTCTCCGAGGAGAAACA-3′
	R: 5′-AAAAACAGCTCCGCATCAAC-3′
Sox-9	F: 5′-GTACCCGCACTTGCACAAC-3′
	R: 5′-TCTCGCTCTCGTTCAGAAGTC-3′
RHAMM	F: 5′-GGCTGGGAAAAATGCAGAGGATG-3′
	R: 5′-CCTTTAGTGCTGACTTGGTCTGC-3′
TLR-2	F: 5′-ATCCTCCAATCAGGCTTCTCT-3′
	R: 5′-GGACAGGTCAAGGCTTTTTACA-3′
TLR-4	F: 5′-CCCTGAGGCATTTAGGCAGCTA-3′
	R: 5′-AGGTAGAGAGGTGGCTTAGGCT-3′
TSG-6	F: 5′-AATACAAGCTCACCTACGCAG-3′
	R: 5′-GGTATCCAACTCTGCCCTTAG-3′

## Data Availability

Not applicable.

## Abbreviations

BMP-2	bone morphogenetic protein-2
BMSCs	bone marrow-derived mesenchymal stem cells
BSA	bovine serum albumin
Cox-2	cyclooxygenase-2
ECM	extracellular matrix
EU	Endotoxin unit
EVs	extracellular vesicles
FCS	fetal calf serum
FGF	fibroblast growth factor
GAG	glycosaminoglycan
IL-1β	interleukin-1beta
IL-6	interleukin-6
HA	hyaluronan
HAS	hyaluronan synthase
HMWHA	high-molecular-weight hyaluronan
Hyal2	hyaluronidase 2
MMP	matrix metalloproteinase
MSCs	mesenchymal stem cells
OA	osteoarthritis
PCR	polymerase chain reaction
RHAMM	receptor for hyaluronan-mediated motility
SD	standard deviation
TLR	Toll-like receptor
TSG-6	tumor necrosis factor-stimulated gene-6
